# Synovial sarcoma: Magnetic resonance and computed tomography imaging features and differential diagnostic considerations

**DOI:** 10.3892/ol.2014.2774

**Published:** 2014-12-05

**Authors:** CHANGHUA LIANG, HUAJIE MAO, JING TAN, YINGHUA JI, FENGXIA SUN, WENGUANG DOU, HUIFANG WANG, HONGPO WANG, JIANBO GAO

**Affiliations:** 1 Department of Radiology, The First Affiliated Hospital of Zhengzhou University, Zhengzhou, Henan 450052, P.R. China; 2 Imaging Center, The First Affiliated Hospital of Xinxiang Medical University, Weihui, Henan 453100, P.R. China; 3 Department of Internal Medicine, The Third Affiliated Hospital of Xinxiang Medical University, Xinxiang, Henan 453003, P.R. China

**Keywords:** computed tomography, magnetic resonance imaging, soft tissue, synovial sarcoma, X-ray

## Abstract

The present study retrospectively examined 24 cases of pathologically confirmed synovial sarcoma and analyzed the clinical presentation and imaging findings in order to explore the imaging features of synovial sarcoma. The majority of the lesions were large (>5 cm; 88%), rounded or lobulated, relatively well-defined tumor masses in the extremities near the joints or deeply located. On computed tomography (CT) scans, the lesions demonstrated intensity signals similar to those of muscle. Six cases exhibited punctate calcification in the periphery of the tumor. On T1-weighted images, the largest lesions of >5 cm, were usually heterogeneous, with a signal intensity similar to or slightly higher than that of muscle. On T2-weighted images, heterogeneous high-intensity or slightly high-intensity signals were observed, with 12 cases exhibiting a high signal consistent with hemorrhage and 12 presenting signals that indicated internal septations. Contrast-enhanced scanning revealed heterogeneous enhancement in the majority of the lesions and no enhancement in areas of cystic necrosis or internal septations. Synovial sarcoma has specific imaging features, and comprehensive analysis of CT and magnetic resonance imaging can improve the accuracy of the pre-operative diagnosis.

## Introduction

Synovial sarcoma originates from mesenchymal tissue that undergoes sufficient differentiation to exhibit the histological appearance of the synovium. Synovial sarcomas constitute 10% of all primary soft-tissue malignant tumors (1,2) and occur in a variety of locations, including the head and neck, retroperitoneum and mediastinum. The majority (80–95%) of the tumors are reported in the extremities, with two-thirds being located in the lower limbs, close to the knees, and being closely associated with the tendon sheath, bursae and articular space. An increasing number of synovial sarcomas have been reported in other locations (1,2).

The lack of characteristic clinical manifestations and specific imaging features leads to a high rate of misdiagnosis for synovial sarcoma (3). The present study retrospectively reviewed 24 cases of pathologically confirmed synovial sarcomas and studied the clinical manifestations, magnetic resonance imaging (MRI) and computed tomography (CT) results to improve the accuracy of the pre-operative diagnosis and enhance the differential diagnosis of synovial sarcoma.

## Materials and methods

In total, 24 cases of pathologically proven synovial sarcoma treated at The First Affiliated Hospital of Xinxiang Medical University (Henan, China) between 2004 and 2011 were included in the present study. The subjects consisted of 15 females and nine males, with a median age of 35 years (range, 22–56 years). The patients typically presented with a palpable mass or pain that had persisted for three weeks up to six years. In total, 12 patients presented with local tenderness upon physical examination and six patients possessed distant metastasis at the time of diagnosis. No redness or swelling was observed in any patient.

In total, 18 patients underwent MRI, including 15 who underwent contrast-enhanced MRI. Nine patients underwent CT and another nine patients underwent contrast-enhanced CT scans. MRI fast-spin echo T2-weighted imaging [T2WI; repetition time (TR), 2,800 msec; echo time (TE), 105 msec], T1WI (TR, 400 msec; TE, 20 msec) and short T1 inversion recovery (TR, 8,000 msec; TE, 204 msec; inversion time, 150 msec) were performed with the GE Signa 1.5T or 3.0T MR scanner (GE Healthcare, Cleveland, OH, USA). The contrast-enhanced T1WI MR imaging was performed with 0.2 ml/kg meglumine gadopentetate (Gd-DTPA; Bayer, Guangzhou, China). Scans on the horizontal, coronal and sagittal planes were recorded.

CT scanning was performed with the GE LightSpeed RT 16 CT Scanner (GE Healthcare). Contrast-enhanced CT scans were performed in the arterial phase using non-ionic iodine (Jiangsu Henghui Medicine Co., Ltd., Lianyungang, Jiangsu, China) and contrast medium (concentration, 320 mg/ml), with 1.5 ml/kg being administered intravenously at a rate of 3.0 ml/sec.

Written informed consent was obtained from the families of patients. The study was approved by the ethics committee of The First Affiliated Hospital of Zhengzhou University, Zhengzhou, China

## Results

### Location and size of lesions

Out of the 24 cases of synovial sarcoma, nine were located in the spine, consisting of three sarcomas located in the cervical spine and six in the thoracic spine. Three tumors were located in the ankle, three in the knee, three in the subclavian area around the shoulder joint, three in the groin area and three at the upper thigh. All the lesions were deeply located, with nine lesions located near the joints of the extremities. The lesions observed were between 6.2 and 15.0 cm in size. Six patients exhibited signs of recurrence at one to two years post-surgery.

### CT findings

**Figure 1:**
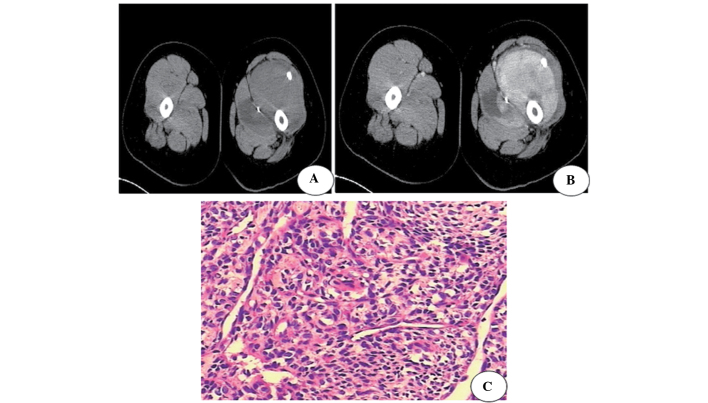
A 31-year-old male with synovial sarcoma. (A) Computed tomography revealing a lobulated tumor mass with a low-intensity signal in the muscle of the left upper thigh. The tumor is well-defined and has punctate calcification. (B) Contrast-enhanced scan revealing heterogeneous enhancement and non-enhancement in areas of necrosis. (C) Pathological confirmation of synovial sarcoma carried out by hematoxylin and eosin staining.

**Figure 2:**
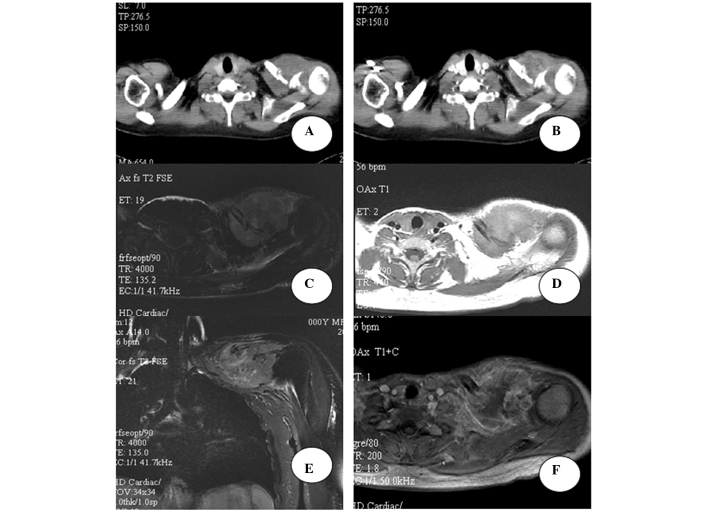
A 27-year-old female with synovial sarcoma. (A–B) Computed tomography revealing a poorly-defined, low-intensity signal tumor mass near the shoulder joint. A contrast-enhanced scan revealing heterogeneous enhancement with osseous destruction of the adjacent clavicle. (C–F) Magnetic resonance imaging revealing a slightly higher-intensity signal relative to muscle on T1-weighted imaging (T1WI) and T2WI. High- and low-intensity signals are also present. Contrast-enhanced scan reveals heterogeneous enhancement with osseous destruction and pathological fracture in the adjacent clavicle.

The images of nine tumors in the shoulder, groin and upper thigh revealed lobulated masses deep in the intramuscular space, with a density slightly lower than that of muscle. Six lesions were unclearly defined and three were well-defined. The masses were heterogeneous, with six lesions exhibiting punctate or lobular calcification (Fig. 1) and six lesions being in contact with the bone and causing osseous destruction (Fig. 2A and B). Contrast-enhanced scans revealed nine heterogeneously enhanced lesions. No enhancement was observed in necrotic or cystic areas (Figs. 1A and B and 2A and B).

### Features of MRI

**Figure 3:**
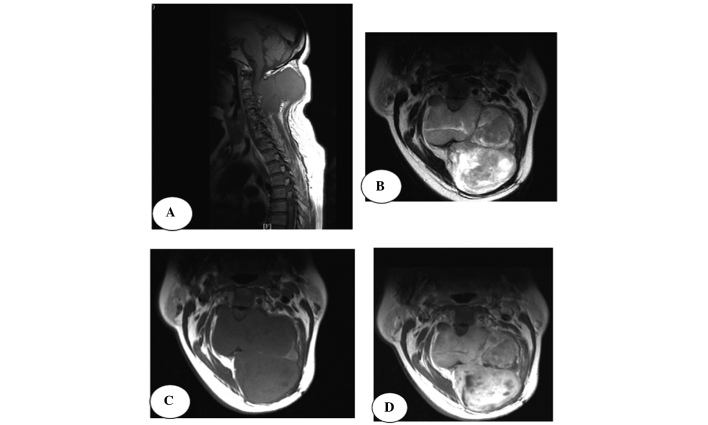
A 56-year-old female with synovial sarcoma. (A) Magnetic resonance imaging revealing a well-defined lobulated soft-tissue mass in the neck. (B and C) T1-weighted imaging (T1WI) and T2-WI revealing a slightly hyperintensive signal relative to muscle and a hypointensitive signal indicating internal septations. (D) Contrast-enhanced scan revealing heterogeneous enhancement, while no clear enhancement is observed in the areas of necrosis and internal septations. Osseous destruction is located in adjacent vertebrae.

**Figure 4:**
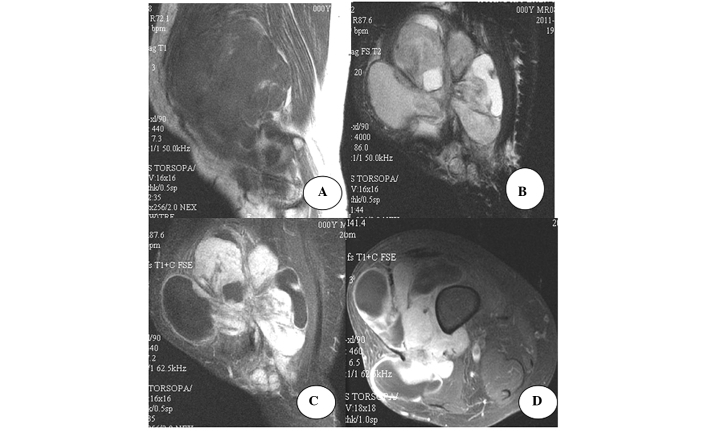
A 51-year-old female with synovial sarcoma. (A–D) Magnetic resonance imaging revealing a well-defined, lobulated soft-tissue mass near the right knee. A slightly high-intensity signal relative to muscle is present on T1-weighted imaging (T1WI) and T2WI. High- and low-intensity signals are also present. Contrast-enhanced scan reveals heterogeneous enhancement, with no clear enhancement observed in areas of necrosis or internal septation.

Images of the 18 lesions located on the spine, knee, ankle and subclavian area close to the shoulder joint revealed that 15 lesions were lobulated masses (Figs. 3 and 4) and deeply located, with 12 well-defined lesions and three unclearly defined round lesions. Overall, 15 lesions caused osseous destruction of neighboring bones (Fig. 3). All the lesions on the spine had compressed the spinal cord. All cases demonstrated areas of hyperintensity, isointensity and hypointensity relative to the muscle. Hyperintensive signals on T1WI and T2WI were observed in necrotic and cystic areas. Nine cases demonstrated a hypointensive T1 signal, suggesting hemorrhage. Three cases with hypointensive T1 signals were considered to possess fibrous tissue. In total, 12 lesions exhibited a hypointensive T2 signal with internal septations. Contrast-enhanced images revealed heterogeneous lesions (Figs. 2–4), with no enhancement in the areas containing cysts, necrosis or septation (Fig. 3D).

## Discussion

Synovial sarcoma is a mesenchymal spindle cell neoplasm, arising from mesenchymal tissues, with the histological appearance of the synovium. Classic biphasic synovial sarcoma exhibits differentiation of the tumor cells into epithelial cells and fibroblasts, and is categorized into three subtypes, including fibroblastic, epithelial and mixed-differentiation tumors (4). The current World Health Organization classification includes synovial sarcomas under the ‘Tumors of Uncertain Differentiation’ classification (5), which accounts for ~10% of all soft-tissue sarcomas (1,2).

Synovial sarcoma is more common in young adults, but can occur at any age, with half of the lesions occurring in individuals between 20 and 40 years old. Males and females are equally affected. Synovial sarcoma is considered the most common malignant non-rhabdomyosarcomatous soft-tissue sarcoma in children and adolescents (6–8). The lesions are commonly located in the extremities, most often in the lower limbs, accounting for two-thirds of synovial sarcomas. Extremity lesions typically occur either in periarticular locations or close to a bursa or tendon sheath. The most common locations are close to the knee and in extra-articular positions, but rarely in an intra-articular position (<10%) (9). Certain studies have demonstrated that these lesions are associated with a trauma that led to injury of the soft tissue around the joint (10). Lesions are rarely observed in the head, neck, mediastinum or peritoneum. Synovial sarcoma grows slowly over two or three years. Patients often present with a palpable, deeply located and painless soft-tissue mass. The lesion generally does not cause significant dysfunction, however, certain patients present with pain, tenderness on palpation and dysfunction of the neighboring joint. In certain cases, pain may be the only symptom in the early stage of the lesion (9). The primary treatment for synovial sarcoma is surgery, which has a 50% post-operative recurrence rate, usually within two years. Overall, ~40% of lesions metastasize to the lungs, bones and lymph nodes (11).

In the current study, nine lesions were located on the spine, which is a rarely affected site according to the literature, indicating that synovial sarcoma may easily be misdiagnosed as another type of tumor, if it is not located in the extremities. Additionally, six patients presented with signs of recurrence within one to two years post-surgery, and six other patients presented with metastasis involving the lung and other sites.

Based on the present results and those reported in the literature, the imaging features of synovial sarcoma can be summarized as follows: Firstly, the lesions are usually in periarticular locations or deeply located within other sites, with a well-defined, round or lobulated soft-tissue mass. Certain lesions present with poorly-defined margins, with a tendency to grow as a diffuse tumor mass along the tendon, tendon sheath and interstitial space, enveloping neighboring tissues and joints, and leading to osseous destruction of the neighboring bones. In general, the size of the lesions tends to be large, with 85% of tumors being >5 cm in size (12). In the current study, all 24 lesions were deeply located within tissues. Morphologically, three lesions were round and 21 lesions were lobulated soft-tissue masses. In total, 15 lesions had well-defined margins, and nine were poorly defined. All lesions were >5 cm in size, and 18 lesions demonstrated neighboring bone destruction.

Secondly, images obtained from CT scans demonstrate areas of isointensity relative to muscle and areas of hypointensity, indicating necrosis and cysts. Areas of calcification are observed in 20–30% of synovial sarcomas (8), with plaque or punctuate calcification mainly located in the periphery of the lesion, termed peripheral calcification. Rare cases of extensive calcification, resembling an osteoid matrix or bone, and a case with a central punctate calcification have been observed. This can aid in differentiating synovial sarcoma from other types of soft-tissue sarcomas (13). In the present study, nine patients underwent CT scans, revealing six tumors with peripheral calcification, six tumors causing osseous destruction of the neighboring bones and three patients who presented with a pathological fracture. CT aids in the identification of subtle soft-tissue calcifications and local bony changes.

Furthermore, MRI is one of the most common imaging examinations for soft-tissue tumors and is considered the modality of choice for the detection and staging of soft-tissue tumors. In the present study, 18 patients underwent MRI examination, revealing 15 tumors that consisted of a heterogeneous mass. T1WI revealed areas of isointensity or hyperintensity relative to muscle in the tumor masses. Areas exhibiting a high signal intensity indicated hemorrhage inside the tumor and a low signal intensity indicated areas of necrosis or calcification. Cystic necrosis has been reported to occur in poorly-differentiated lesions and lesions with a large diameter (14). The tumors in the present study all exhibited a diameter >5 cm and hemorrhage was indicated in 12 cases. Jones *et al* studied the T2WI results of synovial sarcoma in 34 patients and reported that synovial sarcomas were frequently heterogeneous, with a triple-signal intensity that depicted areas of high signal intensity as fluid, isointensity or hyperintensity relative to fat and hypointensity relative to fibrous tissue (15). Combined with pathohistological studies, T2WI reveals that areas of hemosiderosis caused by hemorrhage, calcification and fibrous tissue demonstrate low signal intensity, the solid tumor mass exhibits a slightly higher signal intensity, while areas of necrosis and hemorrhage reveal a significantly higher signal intensity. However, not all synovial sarcoma demonstrate a typical triple signal. In the present study, only 12 cases (50%) exhibited this typical feature, which is consistent with other studies (6,9,15–17). Notably, 18–73% of cases present with fluid-fluid levels, which is considered to be a specific imaging feature of synovial sarcoma (6,8,13,15). On T2WI, 12 cases in the present study exhibited hypointensive internal septation, commonly inside the tumor or in multiple nodules. Although internal septation is not considered to be a specific feature of synovial sarcoma, its presentation with septation often suggests a malignant tumor (18).

Finally, a contrast-enhanced imaging study revealed that tumors of a large size often demonstrate significantly heterogeneous enhanced signals on CT and MRI images, while tumors of a smaller size tend to be homogeneously enhanced (19). Synovial sarcomas in locations other than the extremities have similar imaging features to those in the extremities (20–22). In the present study, 21 cases exhibited significant heterogeneous enhancement, while no enhancement was observed in areas of either cystic necrosis or internal septations. The three patients that presented with significant enhancement similar to that of the arteries were misdiagnosed with aneurysms.

In the literature, all soft-tissue sarcomas, with the exception of liposarcoma, lack specific imaging characteristics (23). The accuracy of the pre-operative diagnosis of soft-tissue sarcomas is only 25% (24). A synovial sarcoma located close to the joint must be distinguished from pigmented villonodular synovitis, which is a slow-growing, homogeneous and well-defined tumor that often exhibits high signal intensity on T1WI and low signal intensity on T2WI, usually homogeneously enhanced and rarely featuring calcification or osteolytic destruction (9). Synovial sarcoma must be distinguished from fibrosarcoma, malignant fibrous histiocytoma, invasive fibroma, leiomyosarcoma and rhabdomyosarcoma (25). Fibrosarcoma often occurs in older patients and tends to be extremely large in size, with less osseous destruction and no significant calcification. Malignant fibrous histiocytoma often occurs in patients aged 50–70 years, is often located in the thigh and is poorly-defined, with a low degree of calcification. Contrast-enhanced scans frequently reveal significant enhancement.

Invasive fibroma most often occurs in middle-aged patients and is frequently located in the thigh, abdominal wall and retroperitoneal space. Generally, these tumors are well-defined and homogeneous, demonstrating hypointensity relative to muscle. On T1WI and T2WI, invasive fibroma tends to present with a low-intensity signal due to the high content of fibrous tissues and is gradually enhanced on contrast-enhanced scans. Leiomyosarcoma is more commonly observed in the uterus and gastrointestinal tract. Necrosis, hemorrhage and cystic change often occur within the tumor. On T1WI, leiomyosarcoma often exhibits the same isointensity as that of muscle. Rhabdomyosarcoma is a common malignant tumor in children and is often a poorly-defined, painless and deeply located tumor mass. On T1WI, the tumor exhibits an intensity similar to that of muscle. Well-defined, slow-growing synovial sarcoma without infiltrative osseous destruction may be difficult to distinguish from benign lesions.

In conclusion, the following characteristics of synovial sarcoma, taken together, may aid in its diagnosis: i) A lobulated or round soft-tissue mass that develops near the joints of extremities, particularly in the lower limbs; ii) the tumor can be well-defined or poorly-defined; iii) the neighboring bones are affected due to infiltration or compression by the tumor; iv) calcification or bone structure can be observed in the periphery of the tumor; v) a high-intensity signal exhibited by the tumor on T1WI, suggesting hemorrhage, and a triple signal demonstrated on T2WI; vi) contrast-enhanced imaging reveals significant heterogeneous enhancement, and areas of cystic necrosis and internal septations can be observed on enhancement; and vii) tumors often occur in young or middle-aged adults. However, due to the rarity of synovial sarcoma in sites other than the extremities, pre-operative diagnosis must rely on other pathological examinations apart from imaging examinations. CT and MRI each have their own advantages in evaluating synovial sarcoma. The combination of the two approaches can improve the accuracy of the pre-operative diagnosis. However, the final diagnosis relies on pathological investigation.
